# A systematic study of critical miRNAs on cells proliferation and apoptosis by the shortest path

**DOI:** 10.1186/s12859-020-03732-x

**Published:** 2020-09-07

**Authors:** Peng Xu, Qian Wu, Deyang Lu, Jian Yu, Yongsheng Rao, Zheng Kou, Gang Fang, Wenbin Liu, Henry Han

**Affiliations:** 1 Institute of computational science and technology, Guangzhou University, Guangzhou, 510006 Guangdong China; 2 School of computer science of information technology, Qiannan Normal University for Nationalities, Duyun, 558000 Guizhou China; 3 College of Computer Science and Artificial Intelligence, Wenzhou University, Wenzhou, 325035 China; 4 Department of Computer and Information Science, Fordham University, New York, NY 10023 USA

**Keywords:** miRNA, Imbalance, Proliferation, Apoptosis, Cell fate gene, Cancer

## Abstract

**Background:**

MicroRNAs are a class of important small noncoding RNAs, which have been reported to be involved in the processes of tumorigenesis and development by targeting a few genes. Existing studies show that the imbalance between cell proliferation and apoptosis is closely related to the initiation and development of cancers. However, the impact of miRNAs on this imbalance has not been studied systematically.

**Results:**

In this study, we first construct a cell fate miRNA-gene regulatory network. Then, we propose a systematical method for calculating the global impact of miRNAs on cell fate genes based on the shortest path. Results on breast cancer and liver cancer datasets show that most of the cell fate genes are perturbed by the differentially expressed miRNAs. Most of the top-identified miRNAs are verified in the Human MicroRNA Disease Database (HMDD) and are related to breast and liver cancers. Function analysis shows that the top 20 miRNAs regulate multiple cell fate related function modules and interact tightly based on their functional similarity. Furthermore, more than half of them can promote sensitivity or induce resistance to some anti-cancer drugs. Besides, survival analysis demonstrates that the top-ranked miRNAs are significantly related to the overall survival time in the breast and liver cancers group.

**Conclusion:**

In sum, this study can help to systematically study the important role of miRNAs on proliferation and apoptosis and thereby uncover the key miRNAs during the process of tumorigenesis. Furthermore, the results of this study will contribute to the development of clinical therapy based miRNAs for cancers.

## Background

MicroRNAs (miRNAs) are small non-coding RNA molecules, they can regulate the expression of most genes in human [1]. The miRNAs can lead to the translation repression or the degradation of their target mRNAs, and thus affect the production of the corresponding proteins [2–5]. One single miRNA can involve in the regulation of multiple genes, and one specific gene can be disturbed by several miRNAs. To study the biological functions of miRNAs in cells, predicting the targets of miRNAs is an important topic. So far, quite a few databases, such as Targetscan [6], miRDB [7], miRanda [8] and mirTarbase [9], have been built to collect miRNA-gene interactions based on biological experiments and/or computation methods.

MiRNAs are extensively involved in a variety of human pathological conditions, such as cancer, Parkinson’s disease, HIV infection, and diabetes [10–12]. For Example, miR-21 could suppress colorectal cancer cells growth by negatively regulating SAV1 while miR-92a could promote cell proliferation, migration, and invasion in hepatocellular carcinoma [13]. To link miRNA with human diseases, the Human microRNA Disease Database (HMDD) curates experiment-supported evidences for human miRNA and disease associations [14]. Currently, the HMDD has collected 35,547 miRNA-disease association entries including 1206 miRNAs and 893 diseases.

Since biological experiment verification is both expensive and time-consuming, some computational methods have been proposed to predict the potential miRNA-disease associations. Peng et al. proposed a rank-based method to detect the differentially expressed miRNAs in individual breast cancer patients [15]. Jiang et al. developed a model to predict the most potential miRNA candidates involved in diseases, by assuming that functional-related miRNAs are more likely to be associated with phenotypically similar diseases [16]. Chen et al. presented a random walk method, RWRMDA, to predict novel miRNA-disease associations based on the miRNA functional similarity network [17].

MiRNA-targeted therapy has become a potential treatment for various complex diseases. For example, SPC3649 is the first small molecule developed targeting miR-122 to treat hepatitis C infection [18, 19]. Streptomycin is another miRNA-targeted drug to inhibit the overexpression of miR-21 in breast, ovarian, and lung cancers [20]. Jamal et al. proposed a method to predict small molecules targeting miRNA by using Naïve Bayes and Random Forest [21]. Qu et al. predicted small molecule–miRNA associations by integrating similar networks between different small molecules, miRNAs, and diseases [22].

In addition, the over- or under-expression of some miRNAs may affect the sensitivity or resistance of some small molecules. For example, let-7b was found to have resistance to cisplatin, which is widely used to treat various cancers including breast, lung, and liver cancers [23]. Some databases, such as ncDR [24], mTD [25] and Pharmaco-miR [26], curated quite a lot resistance-related non-coding RNAs (ncRNA). For example, the current version of ncDR includes 5864 validated relationships between 145 compounds and 1039 ncRNAs (877 miRNAs and 162 lncRNAs). These datasets will help to decipher the molecular mechanism of drug response, screen disease markers in clinical therapy, and promote the targeted research towards ncRNA related drugs.

The inhibition of mRNAs led by miRNAs influences the balance in oncogene and tumor suppressor genes (TSGs) expression in a complex way. Some genes can regulate miRNAs expression by binding to promoter regions; and in turn, the disrupted miRNAs can regulate other genes. These interactions provide a medium for miRNAs exerting epigenetic control upon cell cycle, apoptosis, proliferation and other crucial biologic processes [27, 28]. As the imbalance between proliferation and apoptosis has been known as one of the fundamental features in tumorigenesis [29, 30], understanding how miRNA impacts the two processes can shed light on deciphering tumorigenesis by answering the query: ‘what’s the role of miRNA in tumorigenesis?’ Mendell et al. showed that miR-17 clustering was associated with cell proliferation and cell cycle dysregulation [31]. Chang et al. reported that the P53 gene was able to activate the miR-34 family that affects cell apoptosis and proliferation [32, 33]. Lv et al. demonstrated that miRNA-34a could decrease cells proliferation and chemoresistance by targeting HDAC1 for ovarian cancer [34]. However, these studies only focused on a few individual miRNAs which could not capture their concerted influence in a network view. Recently, Zhou et al. proposed a network model “oncogene–miRNA–TSG network” to study the domino effects of miRNAs on proliferation, apoptosis, and cell cycle [35].

In this study, we proposed a novel miRNA-gene regulatory network (miGRN) approach to investigate the role of miRNAs on proliferation and apoptosis. The proposed miGRN is a cell fate gene regulatory network with their targeted miRNAs. The impacts of miRNAs on proliferation and apoptosis are calculated by their signaling propagation influence by employing the shortest pathways to the downstream cell fate genes. The proposed method provides a more efficient way to determine the impacts of upstream miRNAs on the cell fate genes comprehensively. Our results show that those genes relevant to proliferation and apoptosis are dramatically affected by the differentially expressed miRNAs that may attribute to the imbalance of the two biological processes. Correlation analysis also indicates that the top-ranked miRNAs can exert a negative regulation on the cell fate genes. Our functional analysis shows that the top-ranked miRNAs involve several cell fates related biological processes and interact closely according to their functional similarities. Furthermore, we find that some top-ranked miRNAs may also affect the sensitivity (or resistance) to anticancer drugs. To the best of our knowledge, this is the first systematic investigation of the important role of miRNAs on proliferation and apoptosis. It will shed light on how to determine the critical miRNAs on behalf of proliferation and apoptosis and screen the potential target miRNAs for clinical therapy of cancers.

## Results

### Datasets

#### MiRNA/mRNA expression datasets

We downloaded the MiRNA/mRNA expression datasets of breast cancer and liver cancer from TCGA (http://tcga-data.nci.nih.gov/tcga/). Table 1 lists the number of samples in the two datasets.

**Table 1:** The miRNA/mRNA datasets for breast cancer and liver cancer

Cancer type	miRNA samples (Cancer/Control)	mRNA samples (Cancer/Control)
Breast cancer	1189 (1085/104)	1211 (1098/113)
Liver cancer	424 (374/50)	423 (373/50)

#### MiRNA-mRNA interactions

We first downloaded the miRNA-mRNA interactions from Targetscan7.0 [6] which include 14,441,602 conversed and non-conserved interactions between the 2603 miRNAs and 19,325 mRNAs. To reduce the false-positive relations, we only retain these recorded in at least one of miRDB5.0 [7] and miRanda2010 [8]. Finally, we obtained 2,050,006 miRNA-gene interactions.

### Perturbation on cell fate related genes

**Fig. 1:**
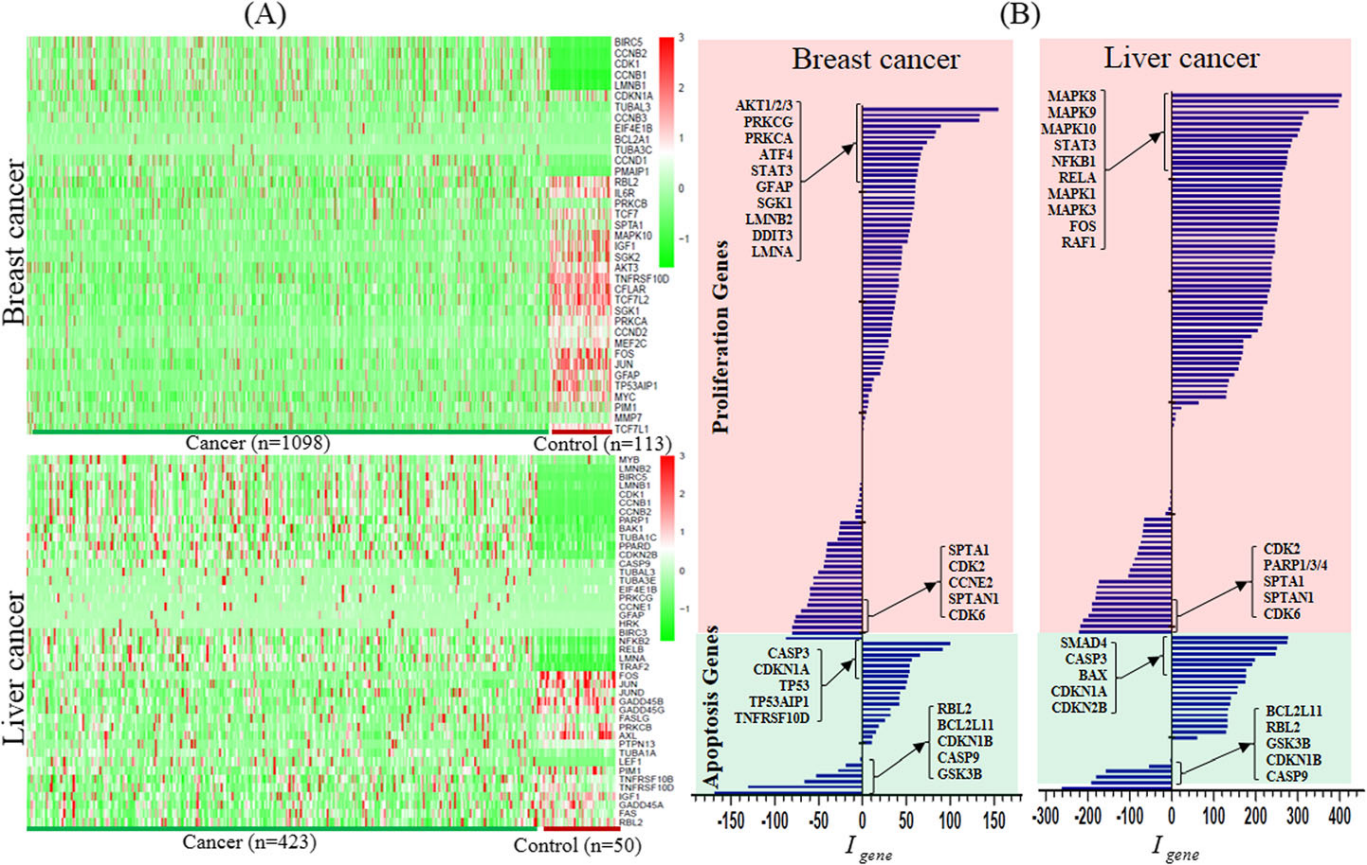
The heat map and global impact of cell fate genes. **a** The heat map of significantly expressed cell fate genes; **b** the global impact on cell fate genes by miRNAs

Figure 1 a shows the heatmap of top ranked differentially expressed cell fate genes. Obviously, some cell fate genes are upregulated while others are downregulated in both breast cancer and liver cancer datasets. It is the abnormal expression of these genes that directly disturb the imbalance of cell proliferation and apoptosis

From the perspective of the miGRN, the abnormal expressions of those cell fate genes are resulted from the abnormal expression fluctuations of the upstream genes and miRNAs. In this study, we just focus on the impact of miRNAs’ perturbation on them. Figure 1b shows the comprehensive influence *I*_*gene*_ of the upstream miRNAs on the 125 cell fate genes in the two datasets. As shown, some proliferation/apoptosis genes are dramatically upregulated/repressed by the upstream miRNAs while others have little influence. The significant impact of these genes by miRNAs is one of the critical driving force to disturb their expressions.

### The critical miRNAs influencing the cell fate genes

**Fig. 2:**
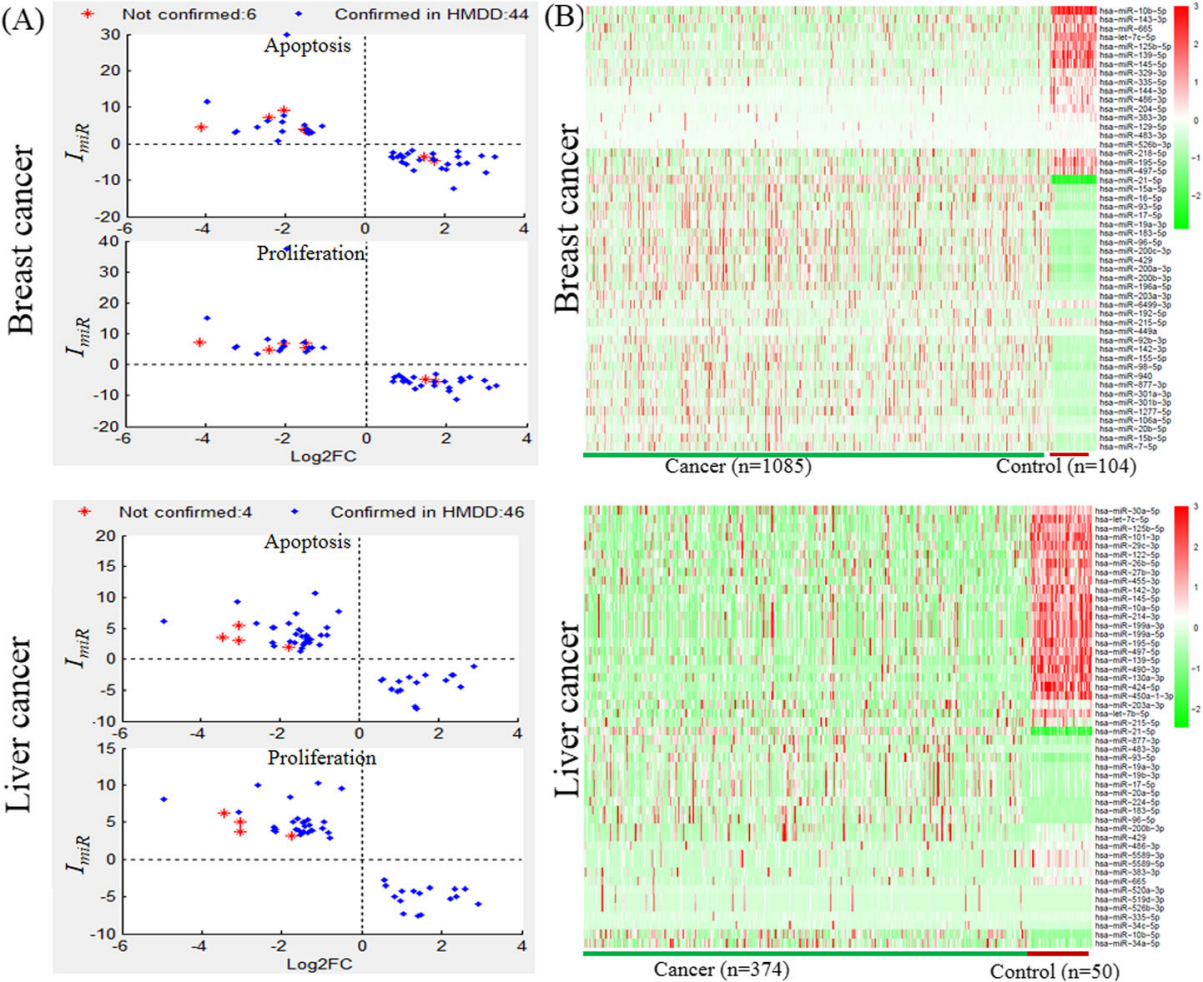
The top 50 miRNAs in breast cancer and liver cancer. **a** The scatter diagram of the average impact on cell fate genes and Log_2_FC; **b** The heatmap of the top 50 miRNAs

In order to investigate the critical miRNAs influencing cell fate genes, Fig. 2a presents the average impact on proliferation genes and apoptosis genes and Log_2_FC of the top 50 miRNAs, and Fig. 2b is the heatmap of miRNAs between cancer and control samples by R program package “Pheatmap”. First, Fig. 2a shows that these miRNAs influence both the proliferation genes and apoptosis genes because of the multiple to multiple regulations between miRNAs and genes. Secondly, the Log_2_FC values of miRNAs are negatively correlated to their total impact on the two kinds of cell fate genes. That is to say, the cell fate genes are generally repressed by the overexpression of miRNAs and activated by their under-expression. Thirdly, 44 and 46 of the top 50 miRNAs are reported to associate with breast cancer and liver cancer respectively in the HMDD database [14]. This demonstrates that the proposed method could identify the critical miRNAs influencing cell fate genes in cancers.

Table 2 lists the top 10 miRNAs in breast cancer and liver cancer. To the best of our knowledge, all the top 10 miRNAs have been demonstrated to be associated with breast cancer and liver cancer via many biological experiments except for miR-665 in breast cancer. Although no evidence supports that miR-665 is breast cancer related, its critical roles in other cancers including liver cancer [36] and pancreatic cancer [37] indicate that miR-665 may be a new potential biomarker or therapeutic target for breast cancer. Besides the miR-665, miR-335 and miR-21 are also the common miRNAs in the top 10 for breast cancer and liver cancer. MiR-335 and miR-21 are confirmed to be associated with advanced clinical stage, lymph node metastasis and patient poor prognosis in both breast cancer and liver cancer [38–40].

**Table 2:** The top 10 miRNAs in breast and liver cancers

Breast cancer				Liver cancer			
Rank	miRNA	log_**2**_FC	PMID	Rank	miRNA	log_**2**_FC	PMID
1	**hsa-miR-335-5p**	−1.97	21,289,068	1	hsa-miR-26b-5p	−1.10	26,891,666
2	hsa-miR-204-5p	−3.97	26,191,195	2	**hsa-miR-335-5p**	−0.67	25,804,796
3	**hsa-miR-21-5p**	2.25	24,980,553	3	hsa-miR-424-5p	−2.61	26,315,541
4	hsa-miR-200c-3p	2.06	22,144,583	4	hsa-miR-195-5p	−1.79	23,468,064
5	hsa-miR-145-5p	−2.44	26,715,279	5	hsa-miR-93-5p	1.41	22,773,266
6	hsa-miR-129-5p	−2.06	22,907,300	6	hsa-miR-490-3p	−4.95	30,007,991
7	hsa-miR-429	3.06	29,702,103	7	**hsa-miR-21-5p**	1.47	23,355,454
8	hsa-miR-155-5p	1.22	22,105,810	8	hsa-miR-483-3p	−3.07	24,127,413
9	**hsa-miR-665**	−2.05	NULL	9	hsa-miR-34a-5p	1.04	28,277,300
10	hsa-miR-200b-3p	2.08	30,342,533	10	**hsa-miR-665**	−1.61	30,237,408

## Discussion

### Biological function analysis of miRNAs

**Fig. 3:**
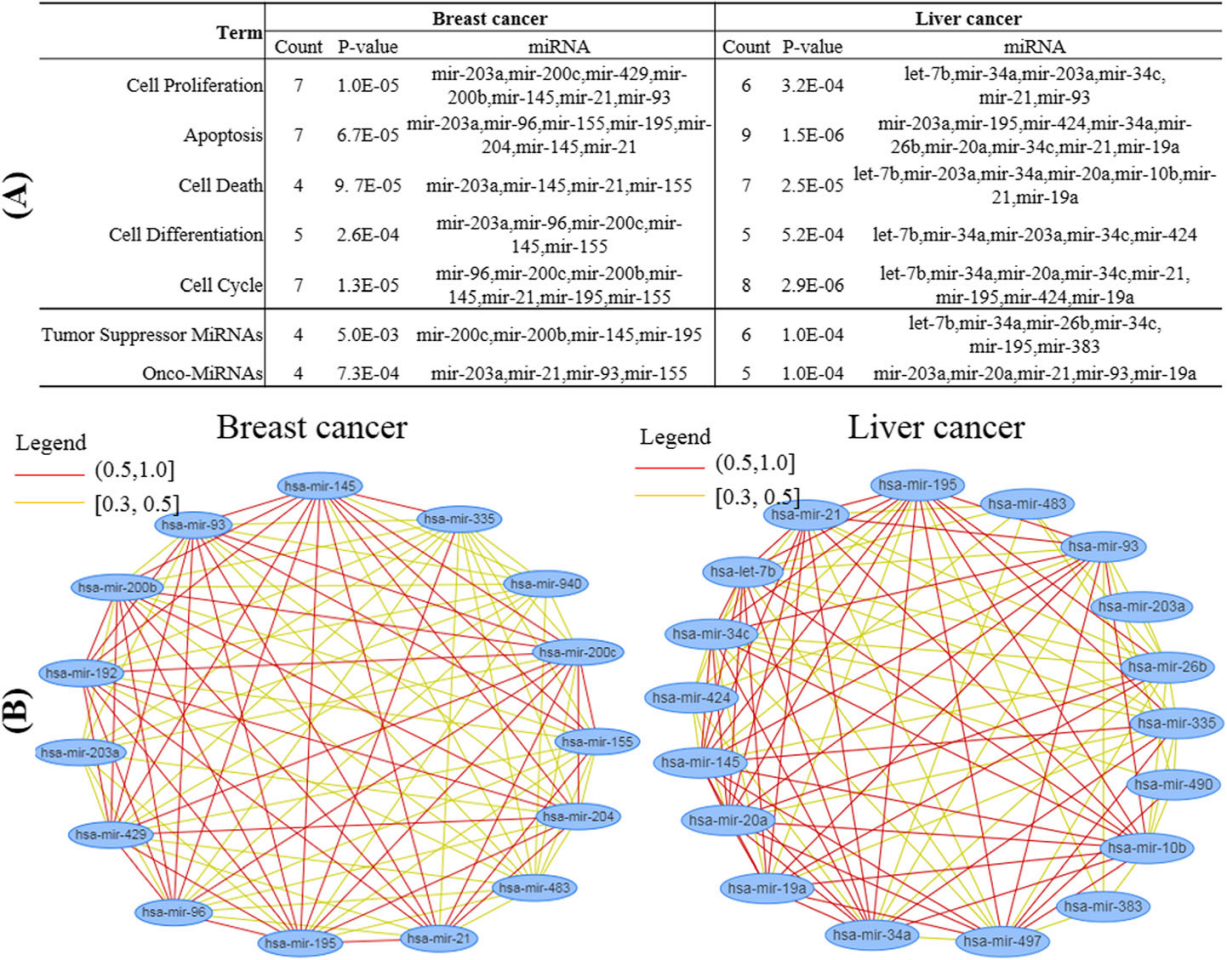
Biological function analysis of the top 20 miRNAs. **a** miRNAs function enrichment analysis; **b** functional similarity network between miRNAs

We further take a biological function enrichment analysis of the top 20 miRNAs on cell proliferation, apoptosis, cell death, cell differentiation and cell cycle by an online tool MISIM [41], which provides functional analysis of miRNAs for complex diseases. Figure 3a shows these miRNAs are significantly enriched in the five functions with *p*-values less than 0.005. More importantly, there are 4(6) onco-MiRNAs and 4(5) tumor suppressor miRNAs for breast cancer (liver cancer) in the top 20 miRNAs. This means that the identified top 20 miRNAs play important roles in cell proliferation, apoptosis and cell cycle and have critical influence on tumorigenesis. We also find some of these miRNAs involved in many other important biological functions, such as hsa-mir-21, hsa-mir-203a, and hsa-mir-145.

MISIM also integrates the co-expression similarity, co-GO similarity, co-literature similarity, and co-similar disease similarity. Figure 3a shows the comprehensive function similarity network of the top 20 miRNAs by MISIM. These miRNAs are closely correlated with an average degree larger than 11, and many of the correlation coefficients are larger than 0.5 (red color). The function similarity network further reveals these miRNAs might interact in a highly coherent way to disturb the proliferation and apoptosis functions.

### Survival analysis of the critical miRNAs

**Fig. 4:**
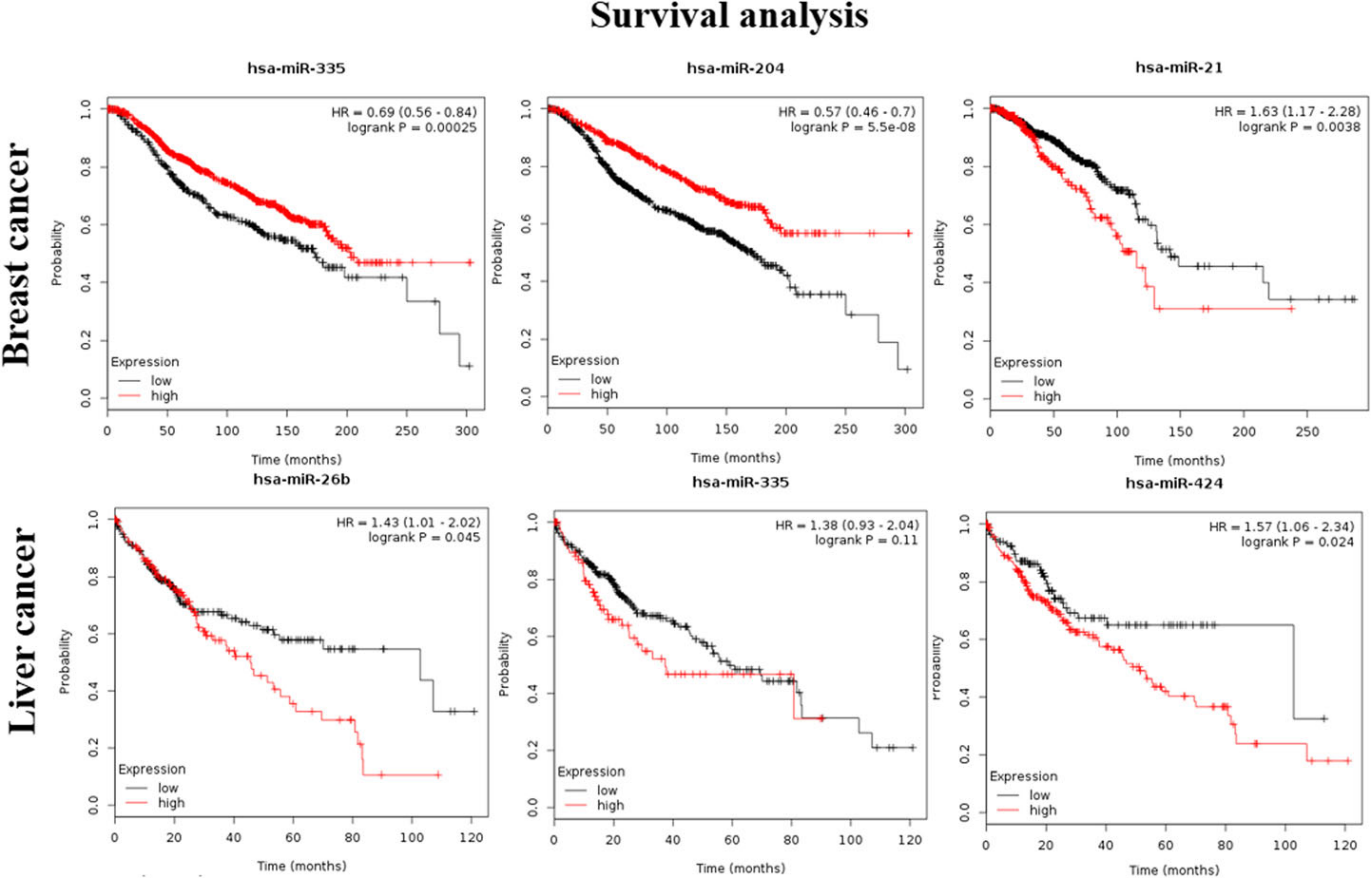
Survival analysis of the top three miRNAs in breast cancer and liver cancer

The validation of prognostic biomarkers is an important clinical issue. Lanczky et al. developed an integrated online bioinformatics tool miRpower to validate the prognostic relevance of miRNAs in various cancers including breast and liver cancers [42]. To evaluate the prognosis performance of the top-ranked miRNAs by our method, we submit the top 3 miRNAs respectively in breast cancer and liver cancer to the online tool. The results show that all the top 3 miRNAs in both cancers are significantly correlated to the overall survival time except for hsa-miR-335 in liver cancer (Fig. 4). This demonstrates that using our method is an effective way to find cancer prognostic biomarkers.

### Drug sensitivity/resistance analysis

Because of the regulation of miRNAs to some important drug targets, it has been proved that some noncoding RNAs (ncRNAs) including miRNAs are able to promote sensitivity or produce resistance on some drugs. Among the top 50 miRNAs, 30 miRNAs can affect drug sensitivity/resistance for breast cancer, and 29 miRNAs for liver cancer. Therefore, these top miRNAs not only play an important role in the dysregulation of cell fate genes but also influence the effect of anticancer drugs. The detailed effects of these miRNAs are included in the supplementary file.

**Fig. 5:**
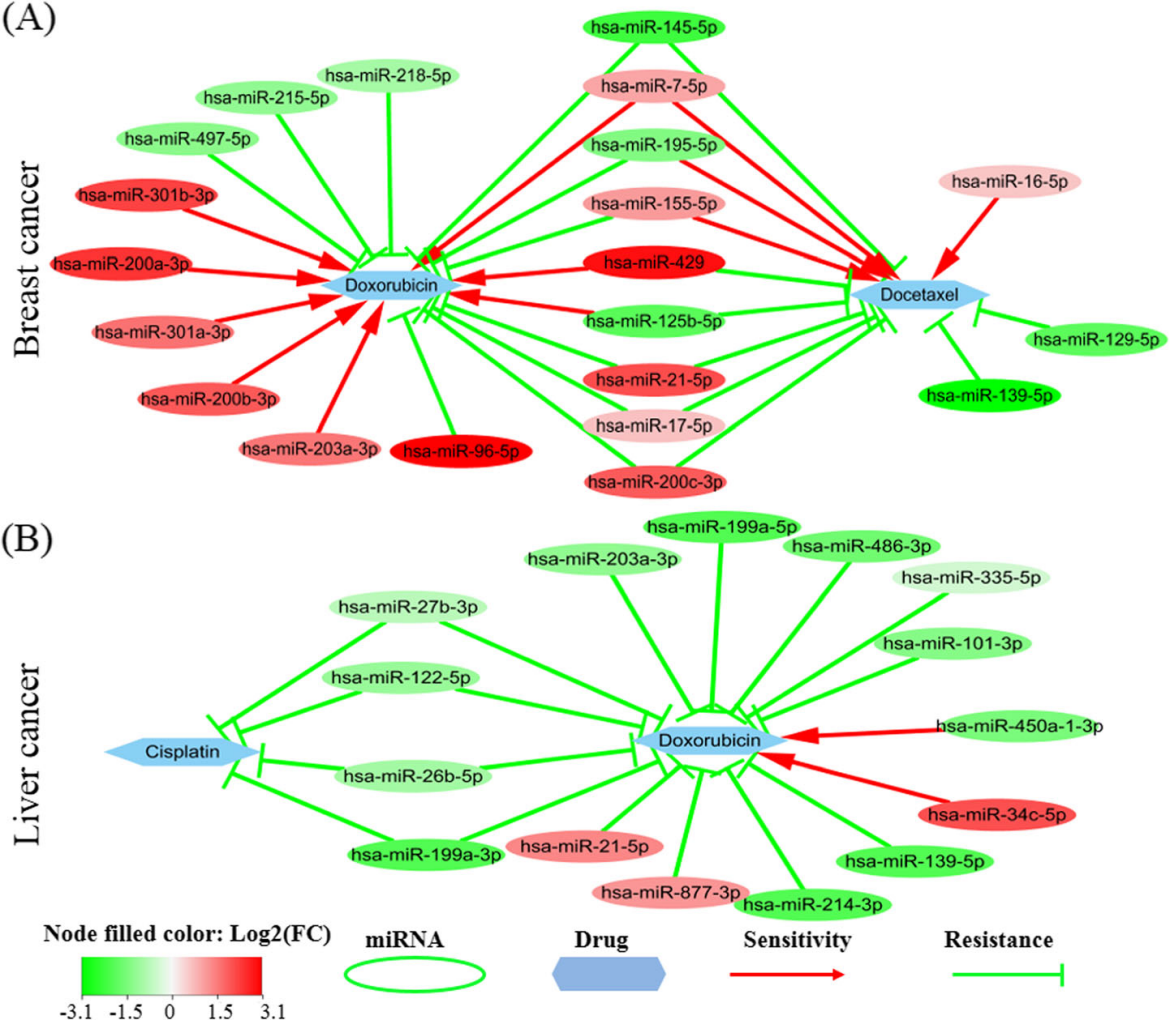
Drug sensitivity/resistance analysis. **a** Docetaxel and Doxorubicin in breast cancer; **b** Cisplatin and Doxorubicin in liver cancer. The red (or green) oval represents the miRNA is up-regulated (or down-regulated) in cancer samples. The red line denotes promoting drug sensitivity while the green line denotes inducing drug resistance

Doxorubicin, Cisplatin, and Docetaxel are three common clinical chemotherapy drugs used to treat many cancers including breast cancer, liver cancer, and stomach cancer. Doxorubicin is an anthracycline, it can slow or stop the growth of cancer cells by blocking an enzyme called topoisomerase 2. Usually, the combination of Docetaxel and Doxorubicin is used as first-line chemotherapy for breast cancer [43]. Cisplatin kills the fastest proliferating cells via interfering with DNA replication. In ncDR, the three drugs are influenced by a relatively large number of critical miRNAs. Figure 5 shows their abnormal expression and influence on drug sensitivity/resistance, which indicates that these miRNAs affect the sensitivity/resistance of drugs in a complex way. Some may promote the sensitivity of a drug while others may induce resistance to a drug. And one miRNA may have different effects on different drugs, such as hsa-miR-429, hsa-miR-155-5p, and hsa-miR-155-5p. Consideration of these miRNAs impact on the drug may help to design a more efficient strategy in clinical treatment. For example, the intervention of the most critical miRNA by either miRNA mimics or inhibitor will both alleviate its dysfunction on cell fate genes and promote the sensitivity of a drug.

## Conclusions

One fundamental principle of tumorigenesis is the dysfunction of the cell cycle, especially, the balance between proliferation and apoptosis is destroyed. As an important regulator of genes, miRNAs have been proved to be involved in the tumorigenesis of various cancers. In this paper, we propose an efficient miGRN approach to study the impact of miRNAs on the perturbation of proliferation and apoptosis. First, we manually extract 125 cell fate genes (including 97 proliferation genes and 28 apoptosis genes) from the KEGG cancer-related pathways. Then trace back to the upstream of these genes, a cell fate gene regulatory network is constructed. After mapping the differentially expressed miRNAs, we calculate the impact of miRNAs on each cell fate genes by the shortest path.

Our results show that some of the proliferation genes and apoptosis genes are dramatically upregulated/repressed by miRNAs, which contribute to the imbalance of proliferation and apoptosis. Based on the impact of these miRNAs, it is confirmed that 90% of the top 50 miRNAs are verified to be related to the corresponding breast/liver cancer in the HMDD database. MiRNAs function analysis indicates that the top-ranked miRNAs significantly relate to cancer-associated biological processes and interact tightly based on their functional similarity. Survival analysis further reveals that the top 3 miRNAs also highly correlated with the survival time of prognosis. Finally, drug sensitivity/resistance analysis based on the ncDR indicates that these miRNAs not only have a significant impact on the balance of the cell cycle but also impact the efficiency of anticancer drugs. In sum, this study deepens the understanding of the molecular mechanisms of miRNAs underlying tumorigenesis and may promote the study for the prediction, diagnosis, and even therapy of cancer.

We should point out that there are some shortcomings in this study. The assumption of the shortest path influence may be too simplistic for the real complex miRNA-gene regulatory process. In addition, we do not take into account other non-coding RNAs, such as lncRNAs, circRNAs, which have also been proved to play critical roles in gene regulation. All these issues will be considered in our future work.

## Methods

**Fig. 6:**
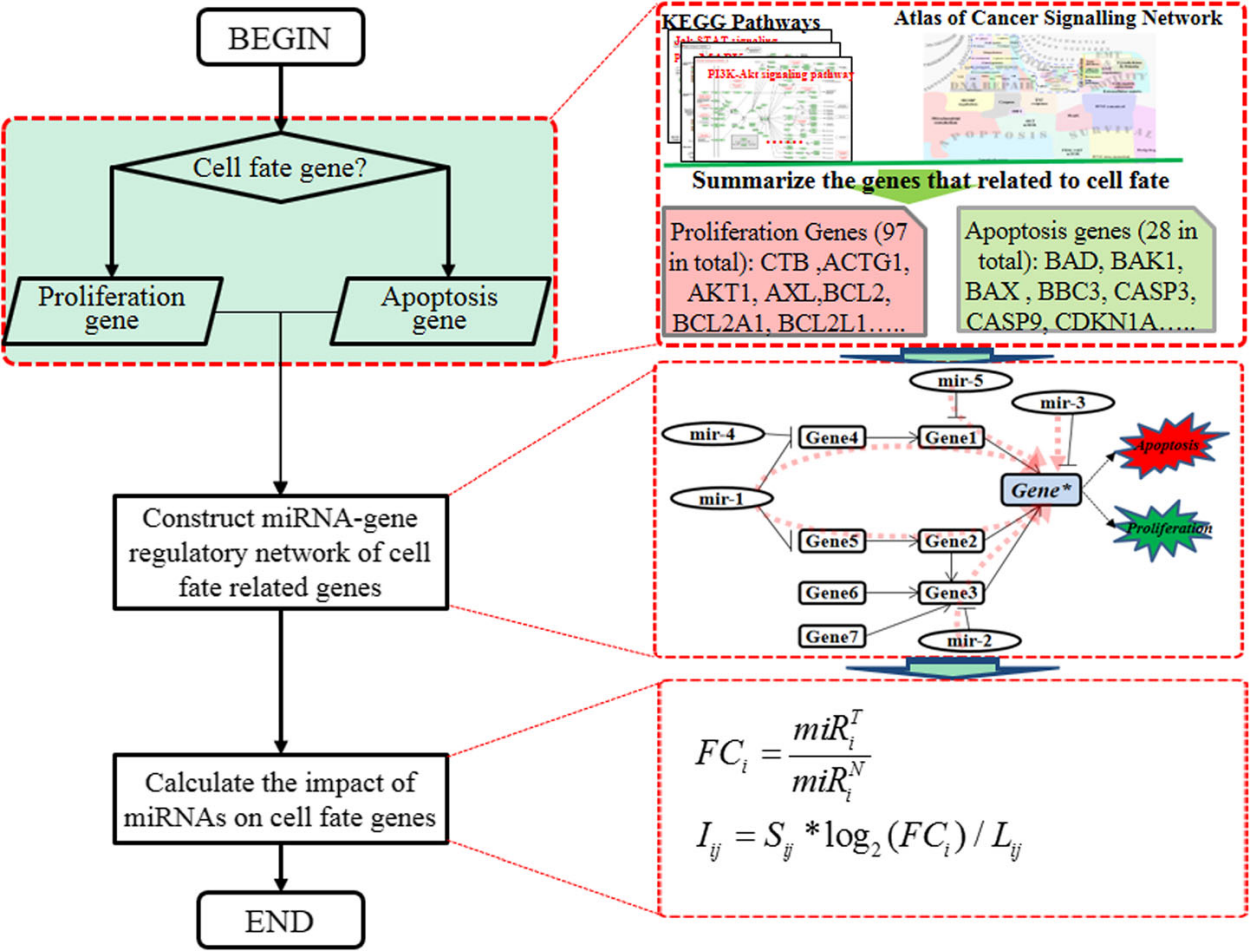
The flow chart of the proposed method

In order to study the perturbation of miRNAs on proliferation and apoptosis, we define the cell fate genes as those directly connecting to biological functions such as proliferation, apoptosis, anti-proliferation, anti-apoptosis, cell differentiation and cell survival in KEGG (Kyoto Encyclopedia of Genes and Genomes) signaling pathway [44]. Specifically, we name the genes relating to the proliferation process as proliferation genes, and those relating to the apoptosis process as apoptosis genes. Then we construct a regulatory network of all the cell fate genes based on the KEGG pathway and map the significantly differentially expressed miRNAs on it based on the miRNA-gene interactions. Motivated by the signaling propagation idea in SPIA [45, 46], we calculate the perturbation of these miRNAs to the downstream cell fate genes by the shortest regulatory paths. Figure 6 shows the main workflow of this study.

### Determine the cell fate genes

**Fig. 7:**
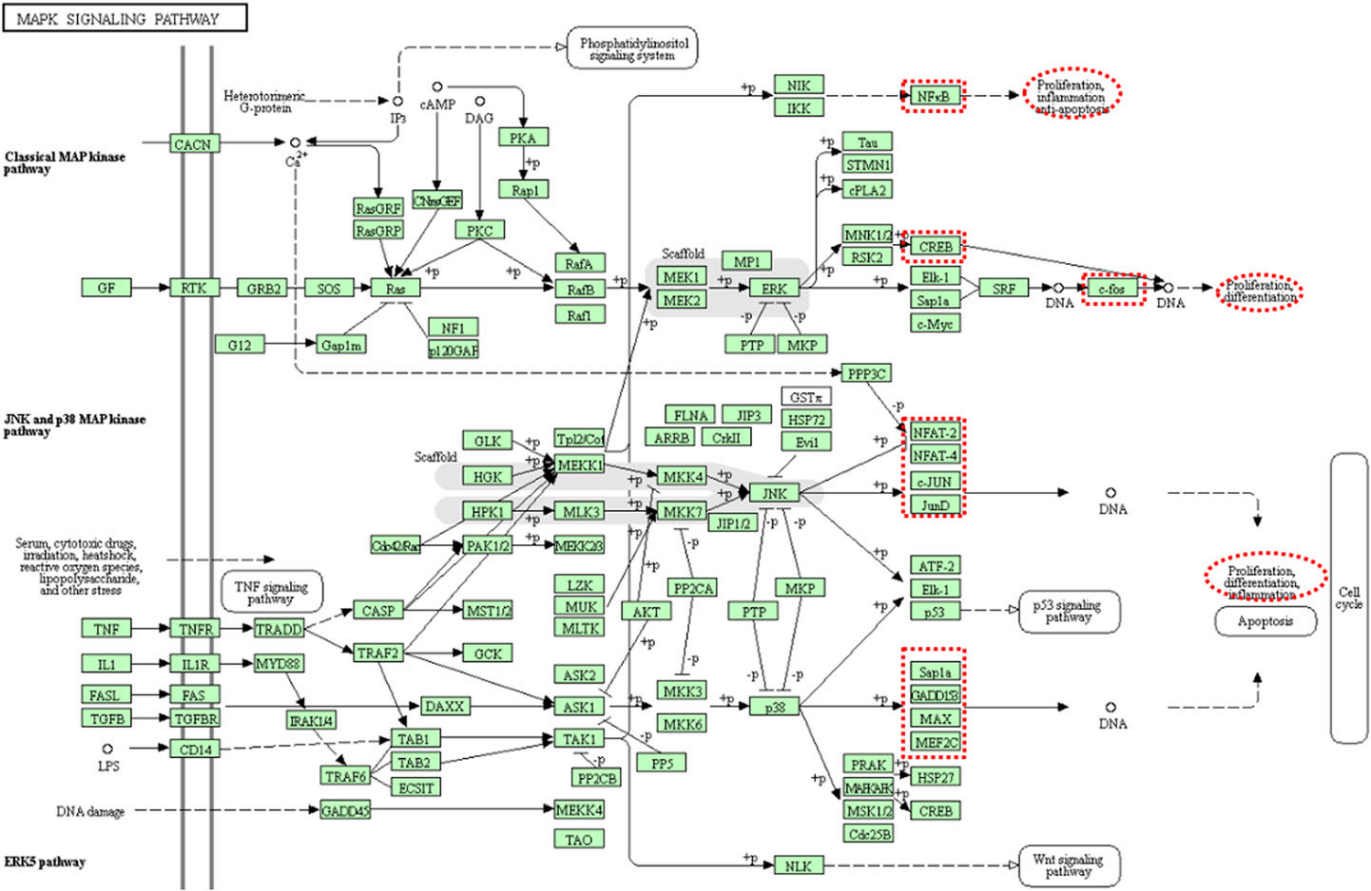
MAPK signaling pathway [44]

Atlas of Cancer Signalling Network (ACSN) is a global biological signaling map [47]. It defines the following 14 pathways as cancer-related signaling pathways: *Apoptosis*, *PI3K-Akt signalling pathway*, *EGFR tyrosine kinase inhibitor resistance*, *p53 signalling pathway*, *Jak-STAT signalling pathway*, *MAPK signalling pathway*, *Wnt signalling pathway, Colorectal cancer, Adherens junction, Focal adhesion, VEGF signalling pathway, cAMP signalling pathway, TGF-beta signalling pathway and Cell cycle signalling pathway*. Then we manually navigate these pathways in KEGG to identify cell fate genes. Figure 7 shows an example of the *MAPK signaling pathway*, where the red ovals are the cell fate functions and the red rectangles point to them are the corresponding cell fate genes. In total, we extract 125 cell fate genes including 97 proliferation genes and 28 apoptosis genes, all these cell fate genes are available in the supplementary file.

### Differential expression analysis

The differential expression analysis of both miRNAs and genes were carried out by an R package “limma” [48]. The *P*-values were adjusted by the FDR method [49]. For the miRNAs or genes with FDR < 0.05 and FC > 1.5 were regarded as significantly differentially expressed miRNAs or genes. Specifically, the significantly differentially expressed cell fate genes are identified from the 125 cell fate genes. In total, we got 558/442 differentially expressed miRNAs and 93/69 differentially expressed cell fate genes for breast cancer and liver cancer respectively.

### Construct the miRNA-gene regulatory network (miGRN) of cell fate genes

The abnormal miRNA signals general transmit in a cascade way from upstream to downstream genes. In order to study their impact on cell fate genes, we construct the miGRN by digging interactions between miRNAs and cell fate genes. We integrated GRAPH and miRNA-target databases to obtain regulatory relations between miRNAs and genes or genes to accomplish it [50]. GRAPH is an R program package to collect well-curated pathway data from authoritative databases such as Reactome [51], KEGG [44] and BioCarta [52]. We first traced the 125 cell fate genes backward based on the regulatory relations in GRAPH to obtain a gene regulatory network related to the cell fate genes. Then, we mapped those significantly differentially expressed miRNAs onto the network to form the final miGRN.

### Evaluate the impact of miRNAs’ perturbation on cell fate related genes

Because of the complexity of gene regulations, the perturbation signaling of one upstream miRNA may propagate to a downstream cell fate gene in many paths. In [45], Tarca et al. proposed a signaling pathway impact analysis (SPIA) to calculate the cascade of perturbation signaling. It integrates the information from classic enrichment analysis with those measuring the actual perturbation on a given pathway under a specific condition. The drawback of this method lies in that it needs complicate computations (e.g. matrix inverse and bootstrap) to model perturbations and assess the significance of observed perturbations. As the perturbation signaling may decay along its flowing path, we assume that the perturbation signaling propagates in the shortest path and it decays linearly with the length of the shortest path. Such an assumption is reasonable because information flow is more likely to follow the shortest path in a biological system given different paths are available for its least cost.

We define the impact of miRNA *i* on the gene *j* as

1 $$ {I}_{ij}={S}_{ij}\ast {\log}_2\left({FC}_i\right)/{L}_{ij} $$

where *FC*_*i*_ represents the average fold change of the miRNA *i* in cancer samples versus control samples, *L*_*ij*_ represents the length of the shortest from miRNA *i* to cell fate gene *j*. The parameter *S*_*ij*_ represents the regulatory mechanism (Promotion/Inhibition) of miRNA *i* to the cell fate gene *j* on the shortest path. *S*_*ij*_ is the product of the regulatory relationship between connected genes on the shortest path. It is noted that we assumed that all miRNAs repress their target genes. As shown in Fig. 6, mir-5 can influence the expression of cell fate Gene* by shortest path “mir (Inhibit,-1) Gene1 (Promote,+1) Gene*”. The *S*_*ij*_ for mir-5 to Gene* can be computed by −1 × 1 =  − 1, which indicates that the expression of mir-5 will inhibit the expression of Gene*.

Given a gene *j*, the overall impact by upstream miRNAs can be calculated as

2 $$ {I}_{gene-j}=\sum \limits_{i\in {R}^j}{I}_{ij} $$

where the *R*^*j*^ is the set of upstream miRNAs on the gene *j*. *I*_*gene* − *j*_ > 0 indicates that the cell fate gene *j* is promoted by its upstream miRNAs and *I*_*gene* − *j*_ < 0 indicates that the cell fate gene *j* is repressed by its upstream miRNAs.

Given a miRNA *i*, its overall impact on all cell fate genes can be calculated as

3 $$ {I}_{miR-i}=\sum \limits_{j\in {G}^i}{I}_{ij} $$

where the *G*^*i*^ is the set of downstream cell fate genes influenced by miRNA *i*. *I*_*miR* − *i*_ > 0 indicates that miRNA *i* promotes the expression of its downstream cell fate genes and *I*_*miR* − *i*_ < 0 indicates that miRNA *i* represses the expression of its downstream cell fate genes.

## Supplementary information

**Additional file 1.**

## Data Availability

Source codes of this study are available at https://github.com/xupeng2017/IMCF.

## Abbreviations

HMDD: Human MicroRNA Disease Database
miRNAs: microRNAs
TSGs: Tumor suppressor genes
miGRN: miRNA-gene regulatory network
KEGG: Kyoto Encyclopedia of Genes and Genomes
ACSN: Atlas of Cancer Signalling Network
SPIA: Signaling pathway impact analysis
ncRNAs: noncoding RNAs
